# Clinicopathological features of 45,X/46,Xidic(Y) mosaicism and therapeutic implications: case report

**DOI:** 10.1590/S1516-31802008000500012

**Published:** 2008-09-04

**Authors:** Henrique Soares, Ana Maia, Miguel Campos, Sofia Dória, José Manuel Lopes, Manuel Fontoura

**Keywords:** Mosaicism, In situ hybridization, fluorescence, Gonadoblastoma, Turner syndrome, Nevus, Mosaicismo, Hibridização in situ fluorescente, Gonadoblastoma, Síndrome de Turner, Nevo epidérmico

## Abstract

**CONTEXT:**

45,X/46,Xidic(Y) mosaicism demands careful and thorough study because of both its variable clinical features and its potential complications.

**CASE REPORT:**

The present case relates to a three-year-old girl with the mosaic karyotype 46,X,idic(Y)(q11.2)[23]/45,X[6]. She had no signs of virilization or Turner's syndrome phenotype, but she was referred to our hospital because she presented reduced growth rate, abnormal facies and a melanotic nevus. After examination, she underwent prophylactic gonadectomy because of the risk of gonadoblastoma. Cytogenetic analysis on the streak gonads and blood showed significant differences in the 45,X cell line between these two tissues. The presence of the sex-determining region Y (SRY) gene did not determine male differentiation, which meant in the present case that the predominance of the X cell line in the gonadal tissue was probably due to the determining factor for female sexual differentiation.

## RESUMO

**CONTEXTO:**

O mosaicismo 45,X/46,Xidic(Y) é uma anomalia genética que exige um estudo minucioso pelas suas variadas formas clínicas e suas potenciais complicações.

**RELATO DE CASO:**

O presente caso clínico refere-se a uma menina de três anos de idade com cariótipo de linfócitos 46,Xidic(Y)(q11.2)[23]/45,X[6], sem sinais de virilização ou estigmas Turner à excepção de má evolução estaturo-ponderal, fácies peculiar e nevo melânico. Estudada, foi submetida a gonadectomia profiláctica por risco de gonadoblastoma. As bandas gonádicas e sangue periférico foram analisados com evidência de uma diferença acentuada no predomínio de linhagem 45,X nos dois tecidos. A presença do gene SRY não se mostrou determinante no sentido da diferenciação masculina pelo que, neste caso, o predomínio da linhagem X no tecido gonadal parece ter sido o fator determinante no sentido da diferenciação sexual feminina.

## INTRODUCTION

Gonadal dysgenesis may result in sexual differentiation anomalies that can be expressed by several genophenotypic forms. In cases without sexual ambiguity, the diagnosis is usually made at puberty. However, growth problems or the presence of minor or major malformations may assist in achieving an earlier diagnosis.

The phenotypes in cases of 45,X/46,XY mosaicism are variable and can show stigmas of Turner's syndrome, mixed gonadal dysgenesis or female or male pseudohermaphroditism.^1,2^ On the other hand, isodicentric chromosomes, which are the most common aberration of human chromosome Y, show a clinical spectrum that can vary from normal male phenotype, with or without infertility, to female phenotype with or without stigmas of Turner's syndrome.^3,4^ The resulting phenotype depends on the proportions of each cell line present and on the locations of the breakpoints in chromosome Y. Fluorescence *in situ* hybridization (FISH) may be an important tool for detecting these cases because it enables detection of cell lines that have low frequencies but are potentially important for sexual differentiation and gonadoblastoma development.^5,6^

## CASE REPORT

The patient was a three-year-old girl who was born at 40 weeks of gestation with a diagnosis of symmetrical intrauterine growth restriction (IUGR). The mother's serological tests were negative. Vaginal delivery took place without any complications. The newborn weight was 2310 g (P5), height was 45 cm (P5) and head circumference was 31 cm (P < 5). The neonatal period was unremarkable.

At the age of eight months, she was admitted to our hospital with a diagnosis of acute pyelonephritis. *Klebsiella pneumoniae* was detected in urine cultures. At that time, she presented a poor growth pattern (P < 3) and a melanotic nevus in the left infrascapular region. Ultrasonography revealed a horseshoe kidney and a renal scintigram (DMSA) gave normal findings apart from the horseshoe malformation. Because of her poor growth pattern (< P3), thyroid function and the presence of antigliadin antibodies were evaluated. It was found that her thyroid function was normal and that antigliadin antibodies were absent. Her psychomotor development was normal and growth was maintained below the third percentile.

**Figure 1:**
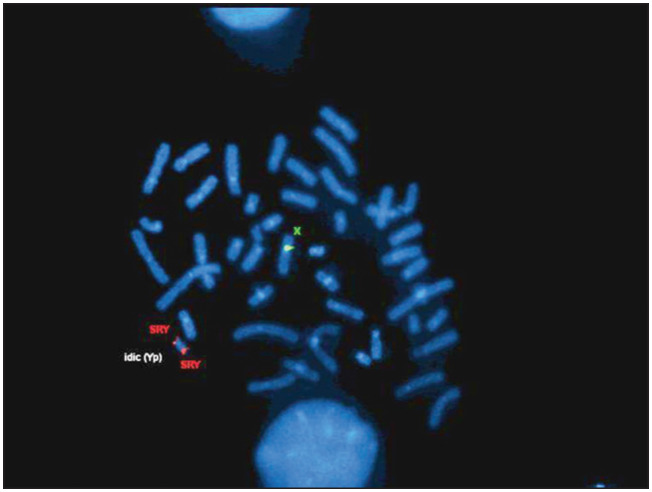
Fluorescence *in situ* hybridization (FISH): sex-determining region Y (SRY) gene.

**Figure 2:**
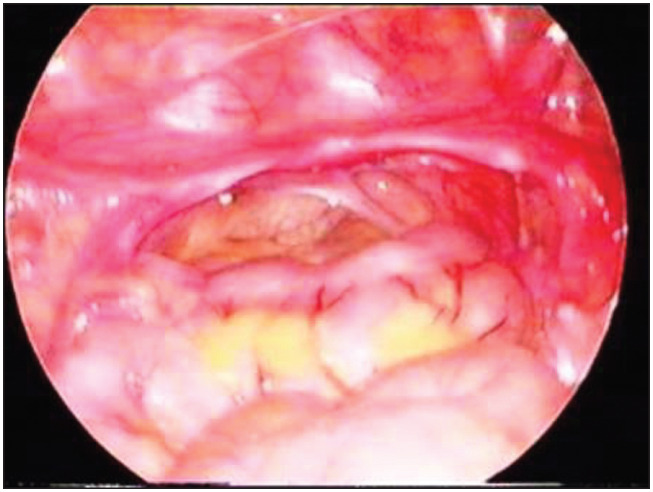
Streak gonads.

**Figure 3:**
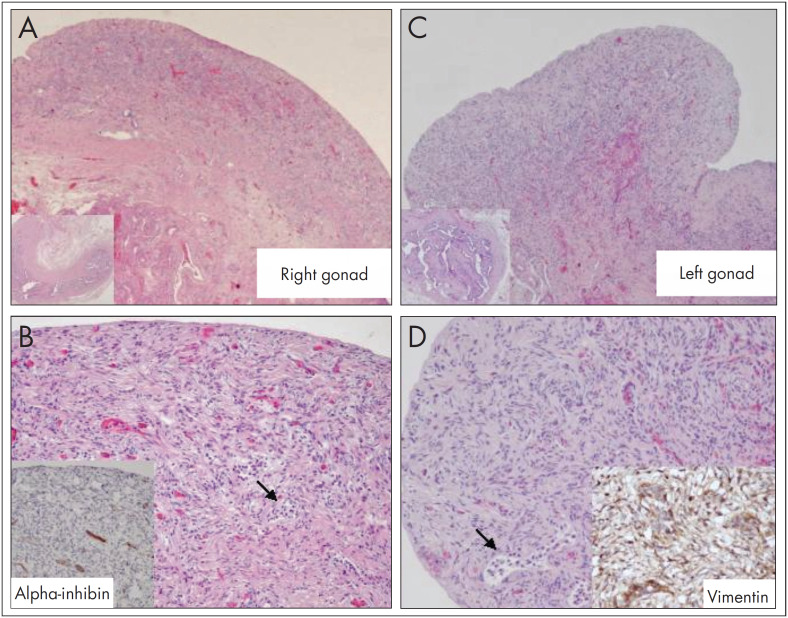
Two streak gonads with the same histological appearance: A. right gonad; B. immunohistochemical localization of alpha-inhibin; C. left gonad; D. immunohistochemical localization of vimentin.

At the age of 29 months, in the light of a poor growth pattern, abnormal triangular facies and the horseshoe kidney, karyotyping was requested. The karyotyping showed 46,Xidic(Y)(q11.2)[23]/45,X mosaicism. Molecular analysis on chromosome Y microdeletions revealed that the azoospermia factor c (AZFc) region and the SRY region were present (Figure 1). Pelvic ultrasonography and abdominopelvic magnetic resonance imaging (MRI) revealed that the Müllerian structures were apparently complete, with no reference to gonadic structures or cross-renal ectopy. Laparoscopy showed macroscopic streak gonads that were asymmetrical, and bilateral prophylactic gonadectomy was performed (Figure 2). Histological examination showed identical gonadic bands with evidence of spindle cell stroma and cordonal structures with cellular expression of inibin α and vimentin, in the absence of CD30 expressing cells (consistent with the morphological absence of gametes) (Figure 3). Fallopian tubes were identified bilaterally. There was no evidence of malignancy. Complementary genetic analysis on the gonadic tissue revealed the karyotype 45,X[221]/46,XX[38]/46,X,idic(Yq)[32]/47,XYY[7]/47,XXY[2], and Turner's syndrome (Turner's mosaic) was diagnosed. Genetic study of the male progenitor gave normal results.

## DISCUSSION

45,X/46,XY mosaicism demands careful study in order to define the sexual differentiation and its therapeutic implications. The present case shows how important karyotyping and physical examination are for analyzing the poor growth rate. The kidney malformation and abnormal facies seen at the age of 29 months required karyotyping analysis, with the aim of early diagnosis. This mosaicism and the phenotype led to a differential diagnosis between mixed gonadal dysgenesis (with a dysgenetic testis and a contralateral streak gonad) and Turner's syndrome.^7,8^ Although the karyotyping studied in lymphocytes showed predominance of the Y line, the female differentiation can be explained by the predominance of the X line in the gonadal tissue.^1,3,9,10^ Today, there is no consensus regarding the need to systematically look for Y material in Turner's syndrome cases with the 45,X karyotype, given the possibility of gonadoblastoma development.^11^ Therefore, FISH takes on a very important role in identifying such cases.^5,12,13^ Although further studies are needed in order to define the precise risk of gonadoblastoma development in cases of 45,X/46,XY mosaicism,^9^ patients with Turner's syndrome or Y mosaicism should have their gonads removed because fertility is not an issue, surgery presents low risk, and there is potentially a high risk of malignant degeneration in gonadic bands.^7,14^
